# Seasonal Analysis of Spatial Distribution Patterns and Characteristics of *Sepiella maindroni* and *Sepia kobiensis* in the East China Sea Region

**DOI:** 10.3390/ani14182716

**Published:** 2024-09-19

**Authors:** Min Xu, Shuhao Liu, Hui Zhang, Zhiguo Li, Xiaojing Song, Linlin Yang, Baojun Tang

**Affiliations:** 1 Key Laboratory of East China Sea Fishery Resources Exploitation, Ministry of Agriculture and Rural Affairs, Shanghai 200090, China; xumin@ecsf.ac.cn (M.X.); zhangh@ecsf.ac.cn (H.Z.); songxiaojing@ecsf.ac.cn (X.S.); bjtang@yeah.net (B.T.); 2 East China Sea Fisheries Research Institute, Chinese Academy of Fishery Sciences, Shanghai 200090, China; 3 First Institute of Oceanography, Ministry of Natural Resources, Qingdao 266100, China; lshuhao666@gmail.com; 4 Xiangshan County Fisheries Bureau, Ningbo 315700, China; lzg4050@sohu.com

**Keywords:** cephalopod, climate change, warm, spawn, nursery, migration, fishery management, SSP1-2.6, SSP5-8.5, resource

## Abstract

**Simple Summary:**

The cephalopod *Sepiella maindroni* de Rochebrune (Hoyle, 1886) is an economically important species in China’s seas, but after habitat loss and overfishing in the 1980s, *Sepia kobiensis* (Hoyle, 1885) gradually replaced its ecosystem roles and fisheries status. Therefore, it is vital to identify the seasonal–spatial distribution variations and characteristics of these two species in recent years. In this study, we found that *S. maindroni* larvae were present at 30.50° N 123.50° E in winter, and the majority of overwintering populations were distributed outside the closed fishing lines of the China seas. Seasonal variations of the recorded sea surface salinity and depth indicated that *S. maindroni* moved from offshore to coastal areas in spring, where they stayed from summer to autumn, and then they migrated to warmer offshore areas in winter. During the spring, we found that *S. kobiensis* tended to be distributed in offshore areas in the northern and in inshore areas in the southern survey areas. We found a greater number of larger individuals in the offshore southern East China Sea in spring and a large number of growing juveniles in summer.

**Abstract:**

Climate change is having important effects on the migration routes and seasonal–spatial distribution patterns of aquatic animals, including the cephalopods *Sepiella maindroni* de Rochebrune (Hoyle, 1886) and *Sepia kobiensis* (Hoyle, 1885) in the East China Sea region. We conducted bottom trawling surveys from 2018 to 2019 in the East China Sea region to identify the seasonal–spatial distribution patterns, including the locations of spawning and nursery grounds of both species, and to determine how they are related to environmental variables. We used random forests and boosted regression trees to identify the distribution patterns of both species from spring to winter to estimate the annual mean situations. We also predicted the habitat distribution variations in 2050 and 2100 under the SSP1-2.6 and SSP5-8.5 climate change scenarios. From our survey data, we detected increasing biomass densities of *S. maindroni* from 29.50° N to 28.50° N, where the largest value of 213.92 g·ind^−1^ occurred. In spring, juvenile groups were present in coastal areas and larger individuals were found in offshore areas. We identified potential spawning grounds at 29.50°–33.00° N 122.50°–123.00° E adjacent to the Zhejiang coastline, and larger individuals and higher biomass densities in south of the 29.50° N line in summer. In autumn, the average individual weight increased in the 28.00° N 122.00° E→124.00° E area. We located potential *S. kobiensis* spawning grounds at 27.00° N 122.00°–123.50° E in spring. Growing overwintering juveniles migrated to the area of 29.50°–30.50° N 125.00°–127.00° E in winter. The sea surface temperature of the areas inhabited by both species showed obvious seasonal variation. The SSP1-2.6 and SSP5-8.5 scenarios indicated that the habitat of *S. maindroni* would shift to the south first and then to the north of the study area with the intensification of CO_2_ emissions, and it would first expand and then greatly decrease. However, the habitat area of *S. kobiensis* would increase. Our results will contribute to a better understanding of the life history traits of both species and the changes in their distribution patterns under different climate scenarios to ensure sustainable exploitation and fisheries management.

## 1. Introduction

Cephalopods are marine invertebrates that typically have a life span of approximately one year [1]. They play an important role in the aquatic food web [2], but they can be strongly affected by environmental and climate changes during their short life history [3]. Therefore, climate change could play a crucial role in the seasonal–spatial habitat distributions of cephalopods. The northwest Pacific Ocean, especially the South Yellow Sea and East China Sea, has the greatest number of cephalopod species and individuals in the world. Among them, members of the family Sepiidae have the widest spatial distribution in this area [4], so it is important to identify the distribution characteristics of the Sepiidae taxa under different climate scenarios because of their important role in the marine environment.

*Sepiella maindroni* de Rochebrune (Hoyle, 1886) and *Sepia kobiensis* (Hoyle, 1885) belong to the *Sepiidae taxa* and are important economic species in China [5,6]. Historically, *S. maindroni*, commonly known as *Moyu* in vernacular Chinese, has been one of the most famous fishery targets in Zhoushan fishing grounds [7]. This species feeds on fish (including *Harpodon nehereus*, *Trichiurus haumela* juveniles, *Setipinna taty*, *Collichthys* spp., *Anguilla* spp., *Cociella* spp., *Engraulis japonicus*, *Jaydia lineata*, *Acropoma japonicum*, and *Johnius belangerii* juveniles) and crustaceans (including *Squilla oratoria*, *Raphidopus ciliatus*, *Acetes chinensis*, *Leptochela gracilis*, *Palaemon gravieri*, and *Solenocera crassicornis*). It is a natural predator of the fish *Larimichthys polyactis* [8,9,10], and adults are preyed on by the fish *Muraenesox cinereus*, *Larimichthys crocea*, *Miichthys miiuy*, *Lateolabrax japonicus*, and adult *T. haumela* and *Epinephelus* spp. [9]. *S. maindroni* prefer to release eggs in areas where they can attach to macroalgae and *Gorgonia* spp. that live around rocky reefs [11]. Fertilized eggs hatch in the subtidal zones in water <20 m deep, and newly hatched larvae and juveniles inhabit the spawning grounds [12].

At water temperature >16.00 °C, the spawning groups of *S. maindroni* belonging to the Huangbohai population may migrate from Huanghai overwintering grounds in May to June to estuarine areas of Huanghe (25.00 °C and 30.06‰) and Laizhou Bay, and to other bays in the Bohai Sea [13]. When water temperatures decrease to <14.00–15.00 °C in November, the recruitment population born in the current year may migrate to the overwintering grounds in the Huanghai Sea [13]. For example, Zhang et al. (1997) found that the *S. maindroni* population in Jiaozhou Bay in the Bohai Sea made spawning migrations in May 1981 when the water temperature was 12.90–15.00 °C, and the newborn population left the spawning and nursery grounds in November 1981 when the temperature was 9.60–14.10 °C (mean, ~11.90 °C) [14]. On the other hand, Tang et al. (1986) found that the overwintering population inhabiting the ~40.00–80.00 m isobath in southeastern Zhejiang areas made spawning migrations to the northwestern sea areas in mid-April of 1984 [15]. They entered the areas of the Nanji and Beiji Islands and the fishing grounds of Pishan, Yushan, Jiushan, Zhongjieshan, and Shengsi in southern Zhejiang, and they spawned in the rocky islands at a depth of ~50.00 m from the end of April to May when the water temperature was 15.00–28.00 °C [15,16]. Larvae and juveniles were found in the coastal areas at 10.00–25.00 m, and the newborn population gradually migrated to offshore areas in September, and made the overwintering migration in November [6].

The annual fisheries production of *S. maindroni* in Zhejiang fluctuated from 20,000 to 70,000 t from 1958 to 1971 [6,7]. It reached 67,000 t in 1959; decreased to less than 20,000 t after 1975; increased to 60,000 t in 1979; sharply decreased to 14,000 t in 1981; fluctuated between 23,400, 20,430, and 21,255 t, respectively, in 1982, 1963, and 1984, and decreased to <10,000 t in 1988 to 1989. The average production was 2500 t in 2006 to 2009 [6]. Due to habitat loss and overfishing, the fisheries production of *S. maindroni* seriously declined in the 1980s, and it was replaced by Sepia species such as *Sepia esculenta* and *Sepia kobiensis* [5]. In the 2010s, the local government in Zhejiang began releasing *S. maindroni* seedlings to restore this fishery resource around the Dongji and Lvhua Islands of Zhoushan, Dachen Island of Taizhou, and Dongtou Island of Wenzhou in each year [17]. At this time, both *S. maindroni* and *S. kobiensis* were the dominant species in the area of 37.00°–38.50° N 118.75°–120.50° E [18]. *S. kobiensis* was the dominant species throughout the year in the western area of 27.00°–34.00° N 127.00° E [5].

Ciannelli et al. (2013) and Puerta et al. (2014) argued that the seasonal–spatial distribution patterns of marine organisms were as important as long-term fluctuations in their abundance [19,20]. Jin et al. (2020) suggested that the spatial distribution of cephalopods is expanding latitudinally [21]. However, little is known about seasonal–spatial distribution characteristics of *S. kobiensis* in the south Yellow Sea and East China Seas. Both *S. maindroni* and *S. kobiensis* are economically and environmentally important, so it is crucial to understand their spatial-seasonal distribution patterns under different environmental conditions.

Jiang et al. (2010) found that under natural conditions, *S. maindroni* adults from Zhejiang released eggs from April to July before they died [22]. However, artificially cultured *S. maindroni* released eggs from April to July (called spring eggs) and from September to November (autumn eggs) [22]. Ni et al. (1985) reported that *S. maindroni* was not a long-distance migration species from south to north but instead was a short-distance migration species from deeper to shallower areas (spawning migration) and then from shallower areas to deeper areas (overwintering migration) [11]. The Zhejiang Inshore Fishery Resources Investigation Committee (1960) reported that *S. maindroni* undertook short-distance migration from south to north and from southeast to northwest [6]. Based on the available historical information, the following questions need to be addressed. (1) After the decline of *S. maindroni* resources over the past 40 years and the continuous release of seedlings in the last 10 years, have the migration routes and seasonal–spatial distribution patterns of this species changed? (2) As the overwintering population of *S. kobiensis* is impacted by the Kuroshio Current, could environmental variations, caused by possible increases in sea surface temperature in winter, affect this population? (3) What effect might climate change have on the seasonal–spatial distribution patterns of *S. maindroni* and *S. kobiensis*? Therefore, the study aimed to identify (1) seasonal–spatial distribution patterns and characteristics, including biomass and densities, of *S. maindroni* and *S. kobiensis*; (2) potential locations of spawning and nursery grounds; (3) ranges of each environmental parameter (high, low) of both species, including sea surface temperature (SST), sea bottom temperature (SBT), sea surface salinity (SSS), sea bottom salinity (SBS), depth; (4) core distribution patterns of both species from spring to winter and the annual mean situation using random forests and boosted regression trees, and (5) variations in the predicted habitat distribution in 2050 and 2100 under the Intergovernmental Panel on Climate Change’s Shared Socioeconomic Pathway (SSP) climate change scenarios SSP1-2.6 and SSP5-8.5 [23]. Our results can be applied to fisheries management actions for the restoration of *S. maindroni* resources and to further a more comprehensive understanding of resource variations under different climate change patterns.

## 2. Materials and Methods

### 2.1. Survey Area and Procedures

**Figure 1:**
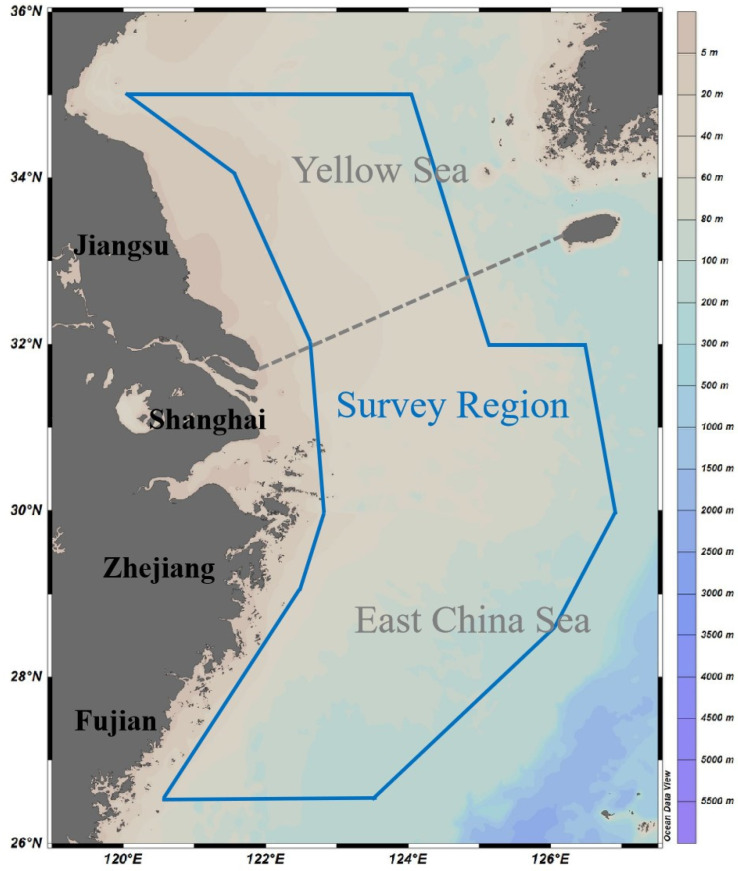
Map showing the survey area (26.50° N–35.00° N 120.00° E–127.00° E), which is denoted by a dark blue solid line border in the East China Sea region, including the southern Yellow and the East China Seas adjacent to the coastline of Jiangsu, Shanghai, Zhejiang, and Fujian. The color bar denotes the depth range from 0 m to 5500 m. The gray dashed line indicates the boundary line between the Yellow Sea and the East China Sea.

Trawling survey data related to *S. maindroni* and *S. kobiensis* were collected from 2018 to 2019 in the southern Yellow and East China Seas (autumn: 1898.19 g·h^−1^ of total CPUE_w_ and 23.30 ind·h^−1^ of total CPUE_n_ from 2–11 November 2018; winter: 1494.83 g·h^−1^ of total CPUE_w_ and 22.88 ind·h^−1^ of total CPUE_n_ from 4–27 January 2019; spring: 929.39 g·h^−1^ of total CPUE_w_ and 9.20 ind·h^−1^ of total CPUE_n_ from 22 April–10 May 2019; summer: 1875.76 g·h^−1^ of total CPUE_w_ and 76.59 ind·h^−1^ of total CPUE_n_ from 13 August to 27 September 2019) [24,25]. We conducted the surveys aboard research vessels (Zhongkeyu 211 and 212) using a trawl net with a headline of 72.24 m, a groundline of 82.44 m, and a cod end mesh size of 20.00 mm [25]. The study areas primarily covered between 26°30′ N and 35°00′ N latitude and 120°00′ E to 127°00′ E longitude, with spaced at longitude 30 min × latitude 30 min intervals using a grid method (Figure 1). We sampled 127 stations in autumn, 111 stations in winter, 141 stations in spring, and 140 stations in summer.

We conducted the following analyses: species identification of the catch in the laboratory to assess the presence of the two cephalopod species at each station; counted and weighed individuals to the nearest 0.1 g of wet weight [26,27,28]; and evaluated catch density per unit of time using two components: biomass density (unit: g·h^−1^) and individual number density (unit: ind·h^−1^). Hydrographic data, including depth, water temperature, salinity, and DO concentration, were collected at each station using a conductivity-temperature-depth profiler (SBE-19; SeaBird-Scientific, Bellevue, WA, USA). We measured SST, SSS, and SSDO content within 3 m below the surface and SBT, SBS, and SBDO content 2 m above the seabed in water shallower than 50 m and between 2 and 4 m above the seabed in deeper water.

The following equations were used to calculate the catch per unit effort (CPUE) by number (n) and weight (w):

CPUE_n_ = *N_i_*/*t_i_*

CPUE_w_ = *W_i_*/*t_i_*

where *N_i_* is the catch in number (ind) at station *i*; *W_i_* is the catch in weight (g) at station *i*; and *t_i_* is the trawling time (h) at station *i*. Additionally, we defined the average individual weight (AIW) as the ratio of CPUE by weight (CPUE_w_) to CPUE by number (CPUE_n_) at a station [24,25].

### 2.2. Modeling

We used machine learning models (random forests (RF) and boosted regression trees (BRT)) to construct the combined model. Both methods were developed to predict aquatic animal habitat distribution range with a reliable forecast performance [29,30]. The RF method is a classifier that contains multiple decision trees and a bootstrap aggregating and bagging ensemble learning algorithm. This model generated multiple decision trees via bootstrap with put-back sampling and finally integrated multiple decision trees to obtain the prediction results. This approach improves the robustness of a single decision tree and has better prediction performance than a single decision tree [31]. The BRT method is based on the idea of a gradient lifting algorithm, which is a model that constructs strong classifiers through the combination of weak classifiers and establishes multiple decision trees in sequence. Each new decision tree performs gradient boosting based on the residual between the predicted value and the true value of the previous decision tree. This method uses the cost function to fit the residual so that each iteration can make the predicted value closer to the true value. When the residual is small enough or the number of iterations is completed, the process stops [32].

Based on the “OptimizeModel” function in “SDMtune” in the R package (version 3.4.6) [33], we used the genetic algorithm of neural network structure to adjust the model parameters of RF and BRT. The genetic algorithm is a method that simulates the evolution process of nature to find the optimal value. It can be used to explore different combinations of model parameters and thus achieve a more comprehensive search and identify the model parameter combination with the highest prediction performance. For the RF and BRT models in this study, 1200 parameter combinations were traversed to construct the species distribution model, and finally the optimal model with maximum area under the receiver operating characteristic curve (AUC) was selected. In the real world, no algorithm can predict species distribution precisely and perfectly. However, the geometric model can combine different algorithms to effectively distinguish noises and capture real signals [34]. Therefore, the ensemble model can effectively improve the accuracy of habitat prediction and reduce bias. The optimal RF and BRT ensemble model was constructed using the “BIOMOD EnsembleModeling” function in “Bimomd2” in the R package (version 3.4.6), which is the package that can estimate relative variable importance using permutation [35].

We assessed the predictive performance of both algorithms using the AUC metric. The AUC is independent of prevalence and is considered a highly effective metric for evaluating a model’s predictive performance [36]. The AUC ranged from 0 to 1, with a value of 1 indicating perfect discrimination and a value of 0.5 suggesting that predictive discrimination is no better than a random guess [37]. In the context of our study, a high AUC value indicates that the model can better distinguish the location of species occurrence and non-occurrence and that the predicted results are more consistent with the actual distribution. In our study, the AUC values obtained using the RF and BRT methods for *S. maindroni* were 0.977 and 0.976, and those for *S. kobiensis* were 0.976 and 0.955. To run the model, we separated the dataset into categories of 0 (absence) and 1 (presence), and then we randomly applied a 70%:30% split for training and testing data independently to develop 10 evaluation runs to construct RF and BRT models using cross-validation [38]. The computation code can be found at http://zenodo.org/records/10408759, accessed on 17 September 2024.

### 2.3. Predictions for the Future

We used environmental data, including SST, SBT, SSS, and SBS, and survey depth from our surveys to represent present-day conditions (2018–2019), which included the spring to winter period and annual mean habitat. We also downloaded modeled future environmental data from the website Coupled Model Intercomparison Project (Phase 6) (CMIP6) to obtain marine data layers (http://esgf-node.ipsl.upmc.fr/projects/cmip6-ipsl/, accessed on 17 September 2024) to explore predicted future variations of habitat distribution in the periods of the 2040s and 2090s and under the SSP1-2.6 and SSP5-8.5 scenarios for both species based on the optimal ensemble model. SSP1-2.6 is a sustainable development scenario that emphasizes sustainability, low resource consumption, low carbon emissions, and a stable radiative forcing of 2.6 W m^−2^ in 2100. In contrast, SSP5-8.5 is a fossil fuel-driven development scenario characterized by high carbon emissions under the assumption that future society will rely heavily on fossil fuels to power economic growth, with a stable radiative forcing of 8.5 W m^−2^ in 2100 [39]. For our 2040s and 2090s case studies, the model used the average values from the IPSL-CM6A-LR (https://www.wdc-climate.de/ui/cmip6?input=CMIP6.CMIP.MPI-M.MPI-ESM1-2-LR.historical, accessed on 17 September 2024) and MPI-ESM1-2-LR (https://www.wdc-climate.de/ui/cmip6?input=CMIP6.CMIP.IPSL.IPSL-CM6A-LR, accessed on 17 September 2024) models of the World Climate Research Programme Coupled Model Intercomparison Project Phase 6 [40,41] to represent future environmental parameters including SST, SBT, SSS, and SBS.

## 3. Results and Discussion

### 3.1. Seasonal and Spatial Distribution Characteristics of S. maindroni

**Figure 2:**
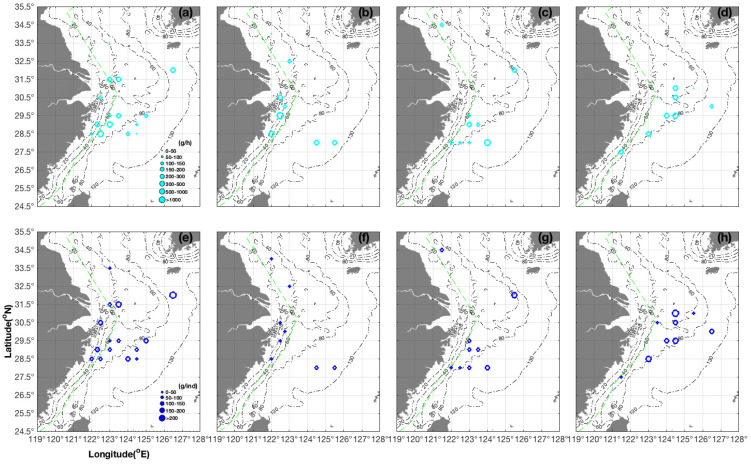
Seasonal distribution characteristics of CPUE_w_ (unit: g/h) shown in cyan (grouped as 0–50, 50–100, 100–150, 150–200, 200–300, 300–500, 500–1000, and >1000 g·h^−1^) and AIW (unit: g·ind^−1^) shown in blue (grouped as 0–50, 50–100, 100–150, 150–200, and >200 g·ind^−1^) for *Sepiella maindroni*. The size of the values is represented by the circles. The depth gradient (20–130 m) is represented by the black dash-dot line. The green dashed line indicates the close fishing lines. (**a**–**d**) CPUE_w_ in (**a**) spring, (**b**) summer, (**c**) autumn, (**d**) winter; (**e**–**h**) AIW in (**e**) spring, (**f**) summer, (**g**) autumn, and (**h**) winter.

For *S. maindroni*, the highest CPUE_w_ values were 160.00, 493.09, and 806.90 g·h^−1^ at 29.50° N 123.50° E, 29.00° N 123.00° E, and 28.50° N 122.50° E, respectively, in the survey latitudinal lines of 29.50° N, 29.00° N, and 28.50° N in spring (Figure 2a). The AIW values varied from 41.81 to 107.00, 56.48 to 122.45, and 65.00 to 107.00 g·ind^−1^, from the inshore to offshore areas along the corresponding survey lines (Figure 2e), which indicated the presence of juvenile groups in coastal waters and larger individuals in the offshore areas. The AIW values were 4.50 and 7.15 g·ind^−1^, respectively, at 34.00° N 122.00° E and 30.50° N 122.50° E in summer (Figure 2f), suggesting potential spawning grounds at 29.50°–33.00° N 122.50°–123.00° E adjacent to the Zhejiang coastline. We recorded the highest CPUE_w_ value of 1111.20 g·h^−1^ at 29.50° N 122.50° E in summer (Figure 2b), so we set a dividing line at 29.50° N. The CPUE_w_ and AIW value ranges were 126.22–214.50 g·h^−1^ and 27.32–33.04 g·ind^−1^, respectively, in the area north of the line at a latitude range of 30.00°–32.50° N (Figure 2b,f), whereas they were 182.77–401.15 g·h^−1^ and 23.40–65.98 g·ind^−1^ south of the line at 28.00°–28.50° N (Figure 2b,f). This result shows that larger individuals and higher biomass densities were present south of the 29.50° N line. The total CPUE_w_ and CPUE_n_ values were 1898.19 g·h^−1^ and 23.30 ind·h^−1^ in autumn, 1494.83 g·h^−1^ and 22.88 ind·h^−1^ in winter, 929.39 g·h^−1^ and 9.20 ind·h^−1^ in spring, 1875.76 g·h^−1^ and 76.59 ind·h^−1^ in summer, respectively.

In the area of 26.50°–30.00° N 121.00°–124.00° E, the AIW value in autumn varied following the order of 23.00, 40.80, 95.00, and 139.28 g·h^−1^ at stations 28.00° N 122.00° E, 122.50° E, 123.00° E, and 124.00° E, respectively (Figure 2g). In the area of 37.33°–38.33° N 118.52°–120.17° E, Wu et al. (1990) found that newly hatched offspring were spawned from August to September [13], but we found that the AIW value in winter was 3.40 g·h^−1^ at 30.50° N 123.50° E (Figure 2h). Wu et al. (2012) reported that spring offspring release occurred from April to May and that the maximum gonadosomatic index values of parent groups in the laboratory occurred in August, with a breeding season from August to October. They also found that autumn offspring release occurred from October to January, the maximum gonadosomatic index values occurred in March, and the breeding season took place from March to May [42]. Cheng et al. (1998) found that parent groups released offspring from March to October, with peak periods from April to June and September to October [43]. In our study, the AIW and CPUE_w_ ranged from 101.30–155.60 g·ind^−1^ and 150.00–311.20 g·h^−1^, respectively, in the area of 28.50°–30.00° N 123.00°–126.50° E in winter (Figure 2d,h), showing that the overwintering populations were present outside the closed fishing lines.

The seasonal orders of average CPUE_w_ and AIW values were summer > autumn > spring > winter and winter > spring > autumn > summer (Table 1), respectively, indicating that the highest average CPUE_w_ value and the majority of juveniles were present in summer, whereas the nursery and overwintering functions took place in the other three seasons. The AIW value ranges indicated the presence of newborn juveniles in summer and winter, and the average AIW values were largest in winter and smallest in summer (Table 1). These results indicated that overwintering parent groups were present in January and released larvae were present in May. We found the largest individuals (213.92 g·ind^−1^) in spring (Figure 2a).

### 3.2. Seasonal and Spatial Distribution Characteristics of S. kobiensis

**Figure 3:**
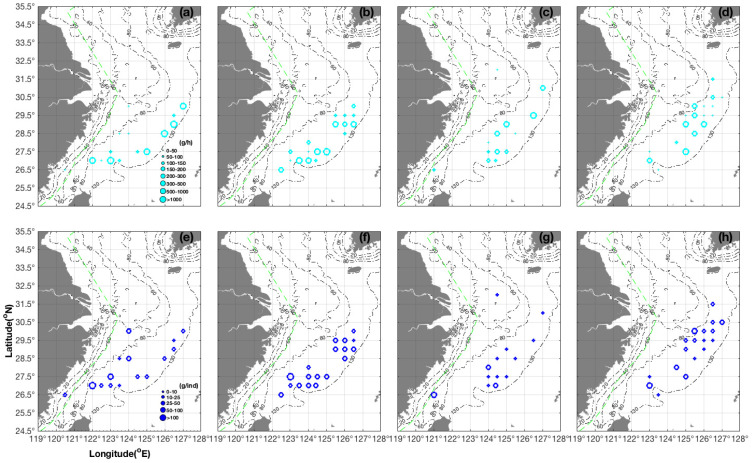
Seasonal distribution characteristics of CPUE_w_ (unit: g·h^−1^) shown in cyan (grouped as 0–50, 50–100, 100–150, 150–200, 200–300, 300–500, 500–1000, and >1000 g·h^−1^) and AIW (unit: g·ind^−1^) shown in blue (grouped as 0–10, 10–25, 25–50, 50–100, and >100 g·ind^−1^) of *Sepia kobiensis*. (**a**–**d**) CPUE_w_ in (**a**) spring, (**b**) summer, (**c**) autumn, (**d**) winter; (**e**–**h**) AIW in (**e**) spring, (**f**) summer, (**g**) autumn, and (**h**) winter.

For *S. kobiensis*, the highest CPUE_w_ values were 626.00, 1779.60, 2079.20, and 3400.00 g·h^−1^ at 30.00° N 127.00° E, 29.00° N 126.50° E, 28.50° N 126.00° E, and 27.00° N 123.00° E, respectively, in the survey latitudinal lines of 30.00° N, 29.00° N, 28.50° N, and 27.00° N in spring (Figure 3a). This pattern indicated a tendency for *S. kobiensis* to be distributed in offshore and inshore areas in the north and south survey areas, respectively. The AIW values varied in the order of 11.18, 15.29, 24.06, and 17.00 g·ind^−1^ at 30.00° N 127.00° E, 28.50° N 126.00° E, 27.50° N 125.00° E, and 27.00° N 123.00° E, respectively (Figure 3e). Thus, more and larger individuals were present in the southern East China Sea, and higher CPUE_w_ and larger AIW values were found in the offshore area (e.g., as shown by the comparison of values between the stations at 28.50° N 123.50° E and 28.50° N 126.00° E (Figure 3a,e). Xu et al. (2024) suggested that the southern East China Sea region was a spawning ground for *Sepia esculenta*, and they found more juveniles in coastal shallow areas and larger individuals in offshore sea areas in spring [24]. In our study, the AIW values of 1.676 and 129.00 g·ind^−1^, respectively, at 27.00° N 123.50° E and 122.00° E (Figure 3e) were indicative of a possible spawning location for *S. kobiensis*. Meanwhile, Xu et al. (2024) reported a possible spawning area of *S. esculenta* at 30.50° N 124.00° to 30.50° N 124.50° E in spring [24].

In summer, most *S. kobiensis* collected in the latitudinal area of 28.50°–30.00° N were juveniles, as indicated by an AIW value of 4.44 g·ind^−1^ at 29.50° N 126.50° E (Figure 3f). Xu et al. (2024) reported that growing juvenile *S. esculenta* might have dispersed widely for feeding and growth along the latitude of 30° N and to the south in summer [24]. In autumn, the majority of *S. kobiensis* AIW values were between 2.38 and 8.80 g·ind^−1^ in the study area (Figure 3g). Growing juveniles at 15.00–17.00 g·ind^−1^ that overwintered migrated to 29.50°–30.50° N 125.00°–127.00° E, and larvae at 1.27–1.50 g·ind^−1^ were found at 29.50° N 126.00°–126.50° E (Figure 3h). These results indicated the presence of potential spawning grounds in the area of 28.50° N to the south and 125.00° E to the east.

The seasonal order for AIW values was summer > spring > winter > autumn, and that for CPUE_w_ values was spring > summer > autumn > winter (Table 1). The lower limit range of AIW values in the four seasons was 1.27–4.44 g·ind^−1^ (indicating new-born larvae), and the upper limit ranges were classified into spring to summer (110.40–129.00 g·ind^−1^) and autumn to winter (66.40–73.50 g·ind^−1^) (Table 1). The total CPUE_w_ and CPUE_n_ values were 2348.96 g·h^−1^ and 495.10 ind·h^−1^ in autumn, 506.46 g·h^−1^ and 204.28 ind·h^−1^ in winter, 9409.49 g·h^−1^ and 650.69 ind·h^−1^ in spring, and 5206.61 g·h^−1^ and 187.96 ind·h^−1^ in summer.

### 3.3. Range of Environmental Variables for S. maindroni and S. kobiensis

SBT is one of the abiotic factors that stimulate the spawning and overwintering migrations of cephalopods [44]. In areas inhabited by *S. maindroni*, we detected an obvious seasonal variation of recorded SST of 14.88–23.60 °C in spring, 25.17–27.50 °C in summer, 19.13–23.08 °C in autumn, and 14.56–17.80 °C in winter (Table 2). Chen et al. (2021) suggested that the most suitable SST values for Sepiidae taxa were 15.10–16.50 °C in spring and 21.07–22.34 °C in autumn [4]. We found that the recorded range of SST and SBT values for *S. maindroni* were similar in winter, and the upper limit values of recorded SST and SBT were similar in summer. The lower limit of SST value was 7.00 °C higher than that of SBT in summer (Table 2), illustrating a large difference in SST and SBT. However, Zhang et al. (2011) reported that the developing biological zero point of fertilized *S. maindroni* eggs was 6.48 ± 0.44 °C, and the effective accumulated temperature during development from the fertilized egg stage to the newly hatched larval stage was 396.91 ± 2.81 °C∙d [45]. They found that the most suitable temperature range for development was 18.00–24.00 °C [45]; the optimal hatching water temperature of wild eggs was 27.00–29.00 °C [22]; and the mean hatching times of wild eggs at temperatures of 27.00–31.00 °C and 17.00–21.00 °C were 16.30–17.70 and 27.80–29.00 days [22]. Additionally, the hatching rate of cultivated eggs was 6.70–30.00% at 19.00–29.00 °C, and incubation failed at >33.00 °C and <17.00 °C [22]. Yin et al. (2005) reported that if larvae were inactive and seldom fed at low temperatures, they were easily preyed upon and attacked by other aquatic organisms [16]. Li et al. (1986) reported that the water temperatures at which eggs attached and in spawning areas were 16.00–25.50 °C and 13.20–22.20 °C, respectively, in the 1950s to 1970s [9].

We classified the recorded SSS values affecting *S. maindroni* into spring to summer (27.86–34.45‰) and autumn to winter (31.88–34.20‰) (Table 2). The recorded ranges of SSS and SBS values were similar in spring and winter, but the lower limit of SBS was higher than that of SSS in summer and autumn (Table 2). The optimal hatching salinity ranged from 24.50 to 32.00‰ and the incubation rates ranged from 18.30% to 25.00% at 19.50–32.00‰; however, hatching rate data were unavailable for salinities <17.00‰ [22]. Li et al. (1986) found that the salinity of the spawning grounds in the coastal areas of the northern Zhejiang for *S. maindroni* was 24.00–29.00‰ during the breeding season [46]. In laboratory experiments, Yin et al. (2005) found that suitable salinity values were 11.73–31.43‰ and 19.61–26.18‰ at water temperatures of 14.00–30.00 °C and 22.00–28.00 °C, respectively [16].

The lower limit of SBDO content was a little higher than that of SSDO in spring, but the ranges were similar in winter. In summer, the SSDO value was in the range of 5.00–6.00 mg/L (Table 2). Li et al. (1986) found that the DO content in the spawning grounds was 7.00–8.80 mg/L [9]. We found that the predicted most suitable depth values were 51.00–74.00 m in spring, 20.00–38.00 m in summer, 15.00–65.00 m in autumn, and 46.00–76.00 m in winter (Table 2). This result shows the movement of *S. maindroni* from offshore to coastal areas in spring to the time duration of summer to autumn, followed by migration to warmer offshore areas in winter.

In areas inhabited by *S. kobiensis*, we also detected obvious seasonal variation of recorded SST values of 16.72–24.56 °C in spring, 26.11–29.19 °C in summer, 20.17–24.82 °C in autumn, and 14.94–22.34 °C in winter (Table 2). Zhu et al. (2014) reported that the habitat of *S. kobiensis* in the southern areas of the East China Sea was mainly impacted by the Kuroshio Current and the western branch of the Taiwan Warm Current [7]. Xu et al. (2024) suggested that the most suitable SBT values for *S. esculenta* from spring to winter were 14.76–20.53 °C, 19.54–22.98 °C, 11.79–17.64 °C, and 16.94–20.36 °C, respectively [24]. In addition, the ranges of recorded SST and SBT values in our study were similar in spring and winter, whereas the recorded SST values were higher than the SBT values in summer and autumn.

The lower limits of recorded SSS from spring to winter were 31.73, 32.55, 32.71, and 33.77‰, and the upper limits ranged from 34.30 to 34.62‰. This result showed that the distribution area moved from inshore to offshore areas from spring to winter. Additionally, the recorded SSS and SBS ranges were similar in autumn and winter (Table 2). Xu et al. (2024) found that the shallower areas were inhabited by *S. esculenta*, as indicated by the SBS values of 31.53–34.80‰, 32.95–34.68‰, 31.51–34.77‰, and 33.82–34.51‰ from spring to winter [24].

The DO ranges were ~5.00–7.00 mg/L in summer and ~7.00–8.00 mg/L in winter, and the ranges of values of SSDO and SBDO were similar in summer and winter (Table 2). Xu et al. (2024) reported that the SSDO values for *S. esculenta* (4.77–6.43 mg/L) were higher than those of SBDO (2.51–6.65 mg/L) [24]. In our study, the predicted most suitable depth values were 113.00–140.00 m in spring, 94.00–133.00 m in summer, 85.00–115.00 m in autumn, and 105.00–145.00 m in winter (Table 2).

### 3.4. Most Suitable Habitat Areas for S. maindroni and S. kobiensis in Present and Future Scenarios

**Figure 4:**
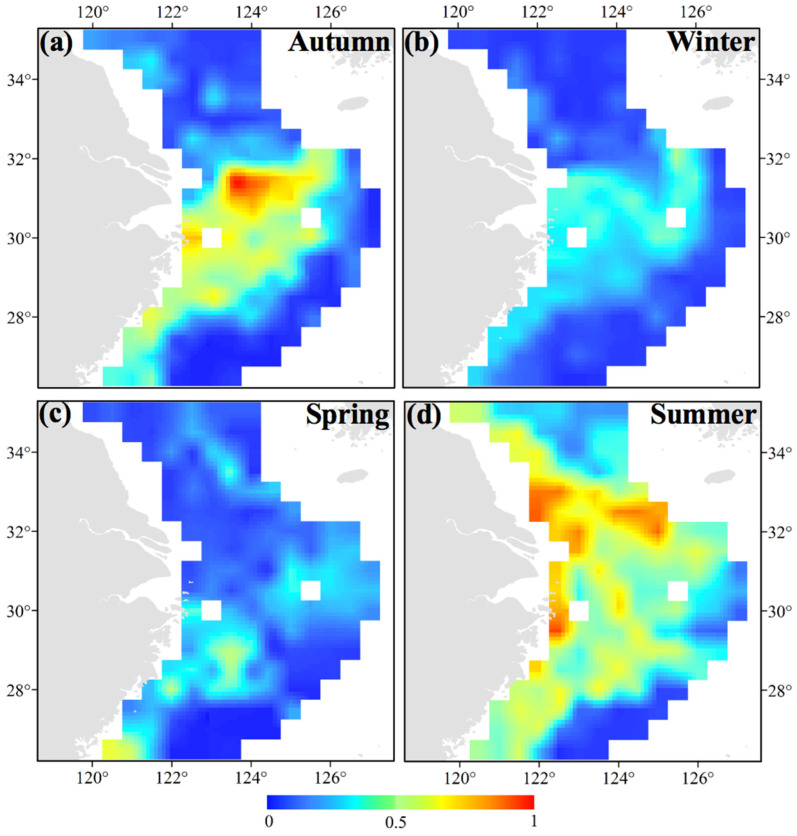
Distribution patterns of *Sepiella maindroni* in the study area predicted using random forests and boosted regression trees in (**a**) autumn in November 2018; (**b**) winter in January 2019; (**c**) spring in May 2019; and (**d**) summer in August 2019. The bar colored in blue to red indicates the range from low to high suitability.

**Figure 5:**
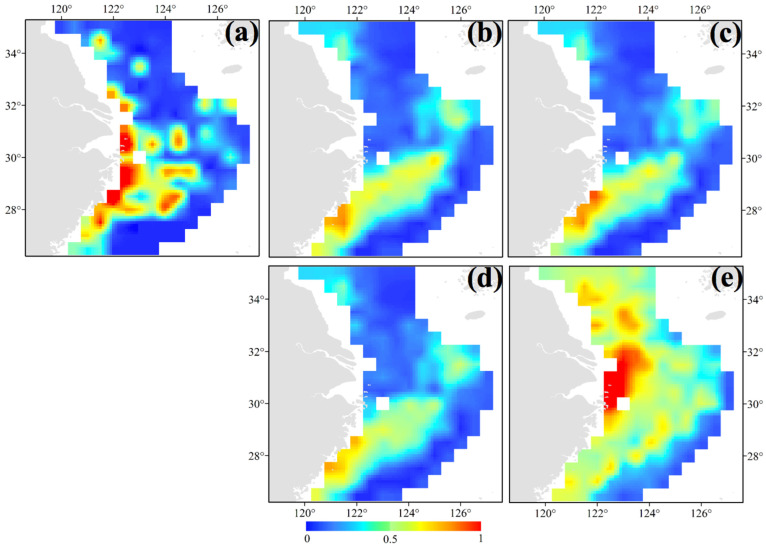
Habitat distribution patterns of *Sepiella maindroni* in the cases of (**a**) annual mean habitat; (**b**) SSP1-2.6 in 2050; (**c**) SSP1-2.6 in 2100; (**d**) SSP5-8.5 in 2050; and (**e**) SSP5-8.5 in 2100. The bar colored in blue to red indicates the range from low to high suitability.

Ni and Xu (1998) argued that the overwintering areas of *S. maindroni* were outside the areas where juveniles feed [11]. Based on our survey data, we found that the majority of groups of *S. maindroni* were in the central and southern areas of the East China Sea in the nearby closed fishing lines, with a most suitable habitat range of 26.50°–29.50° N 120.50°–123.50° E in spring (Figure 4c). Chen et al. (2021) reported that Sepiidae species had similar suitable distribution patterns in the area of 29.50°–31.00° N 122.00°–124.00° E in spring. They also found that the suitable habitat of *S. maindroni* varied from the southwest area of the East China Sea (28.00° N−29.00° N 121.00° E−122.00° E) to the offshore area of the northern East China Sea (30.00° N 123.00° E−125.00° E) in spring [4]. In summer, we found that the majority of *S. maindroni* individuals were concentrated in the southern Yellow Sea in the nearby areas adjacent to closed fishing lines and in areas in and around the Zhejiang Islands (29.50° N−33.00° N 122.00° E−124.00° E) (Figure 4d). This finding suggested a potential relationship between abundance and the release of young, as we observed a large number of juveniles during this season. In autumn, the core distribution areas were in the central and northern areas of the East China Sea off the Zhejiang coastlines in the vicinity of closed fishing lines (30.00°–31.50° N 123.00°–124.50° E); the survey point with the highest density was closest to the Zhejiang Islands (Figure 4a). In winter, the core distribution areas were in the northern East China Sea off the Yangtze Estuary areas extending to the open seas in a limited latitudinal range and in the southwest corner of the survey area along the closed fishing lines off the Zhejiang coastlines (26.50°–32.50° N 121.00°–126.00° E) (Figure 4b). For the annual mean habitat, the most suitable area was 28.50°–30.50° N 122.00°–122.50° E (Figure 5a).

**Figure 6:**
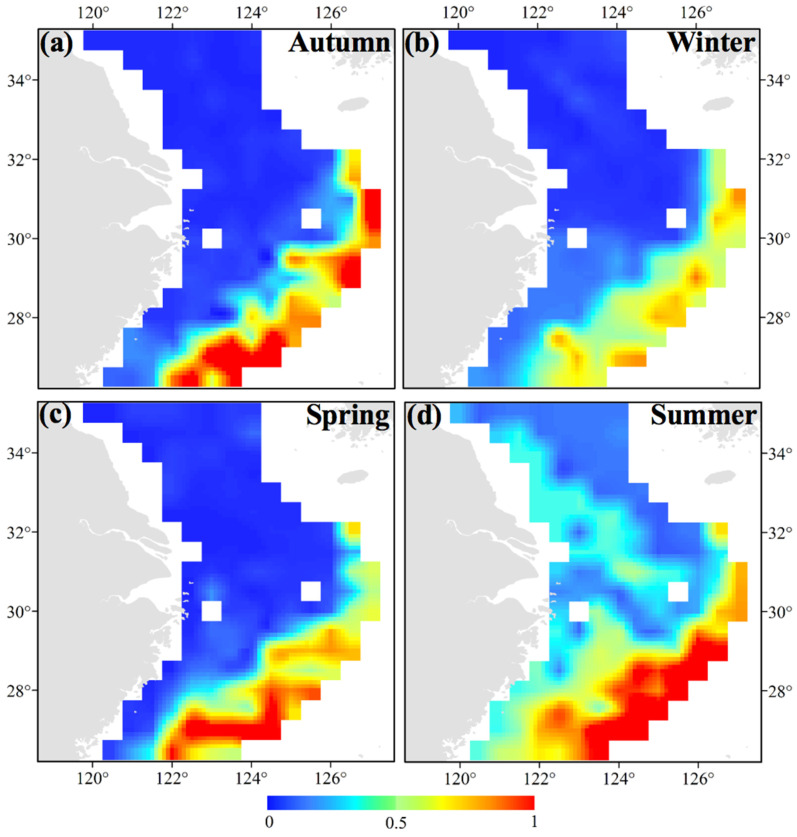
Distribution patterns of *Sepia kobiensis* in the study area predicted using random forests and boosted regression trees in (**a**) autumn in November 2018; (**b**) winter in January 2019; (**c**) spring in May 2019; and (**d**) summer in August 2019. The bar colored in blue to red indicates the range from low to high suitability.

**Figure 7:**
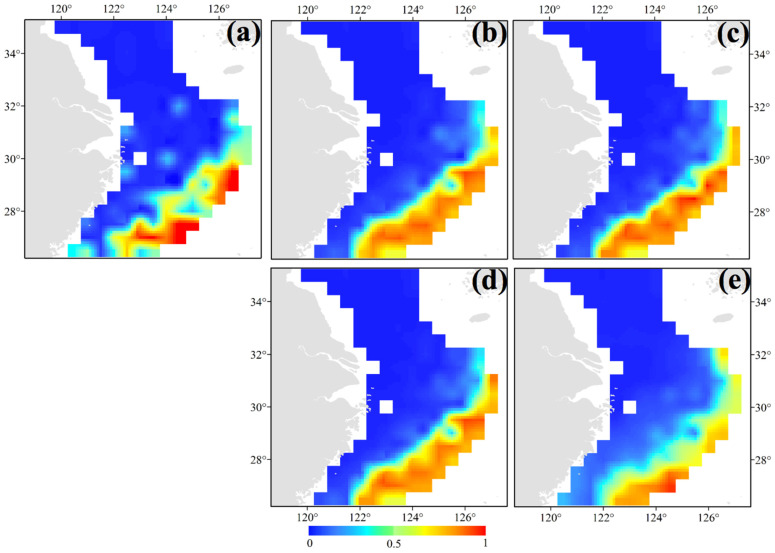
Habitat distribution patterns of *Sepia kobiensis* in the cases of (**a**) annual mean habitat; (**b**) SSP1-2.6 in 2050; (**c**) SSP1-2.6 in 2100; (**d**) SSP5-8.5 in 2050; and (**e**) SSP5-8.5 in 2100. The bar colored in blue to red indicates the range from low to high suitability.

Yan et al. (2007) reported that the central area of *S. kobiensis* distribution was on both sides of the area located at 29.00° N 124.98°–125.43° E [5]. They also found that the distribution showed significant gradient differences in the longitude and latitude directions, with a smaller migration area range compared with that of *S. esculenta* [5]. In our study, the majority of *S. kobiensis* were found in the southern East China Sea, especially in the southeast offshore corner of the survey area with a suitable habitat of 26.50°–27.50° N 122.00°–124.00° E in spring and 26.50°–28.50° N 122.00°–126.00° E in summer (Figure 6c,d). Xu et al. (2024) suggested that the southern area of the Yangtze River to the north was the spawning grounds of *S. esculenta* in spring, and the areas located at 29.00°–34.50° N 124.00°–124.50° E and 28.00°–30.50° N 125.50°–126.50° E were nursery grounds [24]. We found that from summer to autumn, the majority of *S. kobiensis* were distributed in a shallower area with a suitable area of 26.50° N−32.00° N 122.00° E−127.00° E (Figure 6a), but in winter the core areas were reduced to the marginal areas in the central and southern East China Sea, with a suitable habitat of 26.50°–29.00° N 123.00°–126.00° E (Figure 6b). For the annual mean habitat, the most suitable area was 27.00° N−29.50° N 123.00° E−126.50° E (Figure 7a).

For future scenarios of the distribution of *S. maindroni*, we compared the case of SSP5-8.5 in 2050 (26.50°–29.00° N 120.50°–123.00° E) (Figure 5d) with those of (1) the present (28.50°–30.50° N 122.00°–122.50° E) (Figure 5a), (2) SSP5-8.5 in 2100 (30.00°–31.50° N 122.50°–123.00° E) (Figure 5e), and (3) SSP1-2.6 in 2050 (26.50°–30.00° N 120.50°–125.00° E) (Figure 5b). We found that the latitudinal line would move to the south from the present to SSP5-8.5 in 2050, more to the north from SSP5-8.5 in 2050 to SSP5-8.5 in 2100, but that there would be no change between SSP1-2.6 in 2050 and SSP5-8.5 in 2050. When we compared the case of SSP5-8.5 in 2100 with the cases of the present and SSP1-2.6 in 2100 (27.50°–29.50° N 121.00°–124.00° E) (Figure 5c), we found that the latitudinal line would move more to the north with a similar longitudinal area range. Generally, the above analysis indicates that the suitable areas will first shift to the south and then to the north in the latitudes of the study area and that the longitudinal areas will first expand and then decrease as CO_2_ emissions intensify. The habitat area range in the case of slight CO_2_ emissions may always be larger than in the case of heavy emissions (Figure 5a–e).

Based on our survey data, the future scenarios of *S. kobiensis* distribution would involve an increase in the latitude range of the habitats south to 26.50° N and north to 32.00° N, with similar longitude range areas. The model suggested the following ranges for the other scenarios: SSP1-2.6 in 2050 (26.50°–31.00° N 122.00°–127.00° E) (Figure 7b), SSP1-2.6 in 2100 (26.50°–31.00° N 122.00°–127.00° E) (Figure 7c), SSP5-8.5 in 2050 (26.50°–31.00° N 122.00°–127.00° E) (Figure 7d), and SSP5-8.5 in 2100 (26.50°–32.00° N 122.50°–126.50° E) (Figure 7e). These estimates indicate enlarged habitat areas under rising SSTs.

Finally, cephalopod populations such as *Sepia* spp. might have high adaptability to changing climates and environments [47]. Rosa and Seibel (2008) and Hoving and Robison (2012) found that the two cephalopod species of *Dosidicus gigas* and *Vampyroteuthis infernalis* were associated with the oxygen minimum layer, and that both species were physiologically adapted to survive the low oxygen tension of the oxygen minimum [48,49]. Additionally, Gutowska et al. (2008) concluded that cuttlefish were preadapted to ocean acidification [50]. Murphy and Rodhouse (1999) and Hoving et al. (2013) suggested that the characteristics of their life history traits had adapted them for ecological opportunism and provided them with the ability to evolve rapidly under new selection pressures [51,52].

## 4. Conclusions

The main conclusions of this study are as follows.

(1) We found the majority of groups of *S. maindroni* in the central and southern areas of the East China Sea near the closed fishing lines in spring. In summer, they moved to the southern Yellow Sea near the closed fishing lines and Zhejiang Islands, and in autumn they moved to the central and northern areas of the East China Sea near the closed fishing lines. Finally, in winter they migrated to the northern East China Sea off the Yangtze Estuary areas and the southwest corner of the survey area near the closed fishing lines. Generally, they moved from inshore to offshore areas from spring to winter, which was indicated by the SSS index;
(2) Climate change scenarios indicated that the habitat areas of *S. maindroni* will shift to the south first and then to the north of the study area with the intensification of CO_2_ emissions. The habitat area will first expand and then substantially reduce. Generally, the habitat area range in cases of slight CO_2_ emissions may always be larger than in cases of heavy emissions;
(3) In spring and summer, we found the major groups of *S. kobiensis* in the southern East China Sea, especially in the southeast offshore corner of the survey area. In autumn, they were distributed in shallow areas, and finally they migrated to the warmer marginal areas in the central and southern East China Sea in winter. Generally, the majority of groups with large numbers of individuals stayed in the overwintering grounds from winter to spring, but the numbers largely decreased in summer when the adults died after releasing eggs. The number of individuals increased in autumn due to the presence of numerous juveniles. Climate change scenarios showed that the rising SST may result in the enlargement of the habitat of this species.

In future studies, we aim to identify the potential mechanisms that explain why the seasonal variations in CPUE_w_ vary with latitudinal and longitudinal changes related to abiotic factors.

## Figures and Tables

**Table 1:** Seasonal data for catch per unit effort by weight (CPUE_w_) and by number (CPUE_n_) and average individual weight (AIW) for *Sepiella maindroni* and *Sepia kobiensis* from autumn 2018 to summer 2019.

	*Sepiella maindroni*				*Sepia kobiensis*			
	Spring	Summer	Autumn	Winter	Spring	Summer	Autumn	Winter
Mean CPUE_w_ at all stations	21.17	17.78	14.95	13.35	66.26	37.19	18.50	25.05
Mean CPUE_w_ at collection stations	199.02	311.08	210.91	166.09	627.30	289.26	180.69	154.48
Value range of CPUE_w_	22.30–806.90	39.87–1111.20	61.04–835.70	13.60–311.20	13.29–3400.00	19.40–1528.36	27.69–715.29	3.90–672.00
Mean CPUE_n_ at all stations	0.28	0.69	0.18	0.20	4.58	1.34	3.90	3.26
Mean CPUE_n_ at collection stations	2.64	12.13	2.59	2.54	43.38	10.44	38.08	20.13
Value range of CPUE_n_	1.00–12.41	2.77–30.00	1.00–6.00	0.97–9.00	1.00–200.00	1.00–49.09	1.00–150.59	1.00–170.85
Mean AIW	90.41	32.56	86.19	109.35	27.92	31.57	14.45	21.89
Value range of AIW	22.30–213.92	4.50–65.98	23.00–168.57	3.40–276.36	1.68–129.00	4.44–110.40	2.38–66.40	1.27–73.58

**Table 2:** Seasonal in situ ranges of environmental variables in the study area ^a^.

Factor	Spring	Summer	Autumn	Winter
*Sepiella maindroni*				
Depth (m)	19.00–101.00	20.00–108.00	15.00–93.00	46.00–92.00
SST (°C)	14.88–23.60	25.17–27.50	19.13–23.08	14.56–17.80
SBT (°C)	11.56–20.16	18.43–27.79	16.79–21.98	14.64–17.27
SSS (‰)	28.80–34.45	27.86–33.83	31.88–33.99	33.50–34.20
SBS (‰)	28.95–34.77	30.54–34.61	32.00–34.57	33.54–34.37
SSDO (mg/L)	7.84–8.35	5.32–5.92		7.73–8.28
SBDO (mg/L)	8.04–8.84	3.10–6.12		7.81–8.26
*Sepia kobiensis*				
Depth (m)	56.00–116.00	10.00–120.00	41.00–115.00	60.00–145.00
SST (°C)	16.72–24.56	26.11–29.19	20.17–24.82	14.94–22.34
SBT (°C)	15.16–21.93	17.23–28.19	18.26–21.99	15.10–21.55
SSS (‰)	31.73–34.62	32.55–34.30	32.71–34.40	33.77–34.53
SBS (‰)	33.51–35.08	33.68–34.68	32.94–34.71	33.89–34.72
SSDO (mg/L)		5.11–6.54		7.10–8.20
SBDO (mg/L)		4.53–6.60		7.20–8.17

^a^ Abbreviations: SST, sea surface temperature; SBT, sea bottom temperature; SSS, sea surface salinity; SBS, sea bottom salinity; SSDO, sea surface dissolved oxygen; SBDO, sea bottom dissolved oxygen.

## Data Availability

Data are contained within the article.
